# Expanding the Plant GSTome Through Directed Evolution: DNA Shuffling for the Generation of New Synthetic Enzymes With Engineered Catalytic and Binding Properties

**DOI:** 10.3389/fpls.2018.01737

**Published:** 2018-11-30

**Authors:** Evangelia G. Chronopoulou, Anastassios C. Papageorgiou, Farid Ataya, Irini Nianiou-Obeidat, Panagiotis Madesis, Nikolaos E. Labrou

**Affiliations:** ^1^Laboratory of Enzyme Technology, Department of Biotechnology, School of Food, Biotechnology and Development, Agricultural University of Athens, Athens, Greece; ^2^Turku Centre for Biotechnology, University of Turku and Åbo Akademi University, Turku, Finland; ^3^Department of Biochemistry, College of Science, King Saud University, Riyadh, Saudi Arabia; ^4^Laboratory of Genetics and Plant Breeding, School of Agriculture, Forestry and Natural Environment, Aristotle University of Thessaloniki, Thessaloniki, Greece; ^5^Institute of Applied Biosciences, Centre for Research and Technology Hellas (CERTH), Thessaloniki, Greece

**Keywords:** glutathione transferase, directed evolution, DNA shuffling, protein engineering, synthetic biotechnology

## Abstract

Glutathione transferases (GSTs, EC. 2.5.1.18) are inducible multifunctional enzymes that are essential in the detoxification and degradation of toxic compounds. GSTs have considerable biotechnological potential. In the present work, a new method for the generation of synthetic GSTs was developed. Abiotic stress treatment of *Phaseolus vulgaris* and *Glycine max* plants led to the induction of total GST activity and allowed the creation of a GST-enriched cDNA library using degenerated GST-specific primers and reverse transcription-PCR. This library was further diversified by employing directed evolution through DNA shuffling. Activity screening of the evolved library led to the identification of a novel tau class GST enzyme (*PvGm*GSTUG). The enzyme was purified by affinity chromatography, characterized by kinetic analysis, and its structure was determined by X-ray crystallography. Interestingly, *PvGm*GSTUG displayed enhanced glutathione hydroperoxidase activity, which was significantly greater than that reported so far for natural tau class GSTs. In addition, the enzyme displayed unusual cooperative kinetics toward 1-chloro-2,4-dinitrochlorobenzene (CDNB) but not toward glutathione. The present work provides an easy approach for the simultaneous shuffling of GST genes from different plants, thus allowing the directed evolution of plants GSTome. This may permit the generation of new synthetic enzymes with interesting properties that are valuable in biotechnology.

## Introduction

GSTs are multifunctional enzymes that have evolved from a thioredoxin-like ancestor gene (Mannervik, 2012; Labrou et al., 2015). They are involved in different functions such as the detoxification, metabolism, and transport or sequestration of a wide range of endogenous or xenobiotic compounds. GSTs catalyze the nucleophilic attack of reduced GSH (γ-Glu–Cys–Gly) on the electrophilic center of these compounds, leading to the formation of GSH conjugates that display higher solubility and reduced toxicity (Deponte, 2013; Labrou et al., 2015; Perperopoulou et al., 2018).

The majority of cytoplasmic GSTs forms dimers of two identical or different subunits of 23–30 kDa (Labrou et al., 2015; Pégeot et al., 2017). Each subunit displays two ligand-binding sites: a G-site and an H-site. The GSH binds with high specificity to the G-site, which is conserved and is located at the N-terminal domain of the polypeptide. The H-site is the binding site for the electrophilic substrate. It is less conserved and determines the affinity and specificity of GSTs toward the electrophile substrates (Labrou et al., 2015; Pégeot et al., 2017). An induced-fit mechanism has been proposed to facilitate the binding and accommodation of the substrates (GSH and electrophile substrate) to the G- and H-sites (Neuefeind et al., 1997; Axarli et al., 2009a,b).

GSTs are expressed both constitutively and in response to biotic and abiotic stresses including herbicides, herbicide safeners, temperature, chill, drought, light, heavy metals, pathogens, and others (Skipsey et al., 2011; Kissoudis et al., 2015; Islam et al., 2017, 2018; Nianiou-Obeidat et al., 2017; Skopelitou et al., 2017). GSTs are encoded by a large and diverse gene family in plants, which is termed the GSTome. The GSTome differs in the number of GSTs, herbicide specificity, and inducibility in different plants and stress conditions (Liu et al., 2013; Csiszár et al., 2014; Pégeot et al., 2014; Han et al., 2018). Heavy metals and high temperature are also considered as inductors of GST expression and function (Gajewska and Skłodowska, 2008; Wang et al., 2017).

The GSTome consists of the functional GSTs that are encoded and expressed by a genome (Mannervik, 2012). The GSTome comprises the cytosolic, mitochondrial, and microsomal superfamilies. Each superfamily composed by several diverse classes (Nianiou-Obeidat et al., 2017). For example, the cytosolic superfamily in plants has fourteen different classes: tau (U), phi (F), theta (T), zeta (Z), lambda (L), γ-subunit of the eukaryotic translation elongation factor 1B (EF1Bγ), dehydroascorbate reductase (DHAR), metaxin, tetrachlorohydroquinone dehalogenase (TCHQD), Ure2p, and microsomal prostaglandin E synthase type 2 (mPGES-2) (Liu et al., 2013; Lallement et al., 2014a,b). Recently, three new classes were identified in plants: hemerythrin (GSTH), iota (GSTI), and glutathionyl-hydroquinone reductases (GHRs) (Yang et al., 2014). The tau and phi classes have the largest number of GSTs in plants (Liu et al., 2013; Csiszár et al., 2014; Pégeot et al., 2014; Han et al., 2018). Both classes contribute considerable and play key roles in the detoxification of several classes of herbicides (Edwards and Dixon, 2000; Chronopoulou and Labrou, 2009).

The wide catalytic capabilities of GSTs along with their ideal structural features, such as stability, efficient heterologous expression in *E. coli* and purification by a single-step affinity chromatography have encouraged their exploitation in different areas of biotechnology (Perperopoulou et al., 2018). For example, selected GST isoenzymes are being exploited for the assembly of enzyme biosensors, which can find application in the measurements of xenobiotics, such as drugs, toxins, and herbicides (Kapoli et al., 2008; Chronopoulou et al., 2012b; Oliveira et al., 2013; Materon et al., 2014). Furthermore, GSTs have been used in nanobiotechnology for the construction of biochips (Voelker and Viswanathan, 2013; Zhang et al., 2013; Zhou et al., 2014), nanowires and nanorings (Bai et al., 2013; Hou et al., 2013). In plant biotechnology, GSTs are useful tools in plant breeding programs for the development of plant varieties with multiple stresses resistant traits. Alternatively, the use of genetic engineering allows the development of transgenic plants with traits beyond the limitation of the existing genetic variability (Kissoudis et al., 2015; Nianiou-Obeidat et al., 2017). There is, therefore, an urgent need to discover new GST isoenzymes with desired properties for the development of new or novel applications. Protein engineering efforts for the design of new enzymes with improved catalytic and structural properties are required (Broo et al., 2002; Kurtovic et al., 2008; Runarsdottir and Mannervik, 2010).

In the present work, DNA shuffling was employed for the design and creation of a library of tau class GSTs (GSTUs) from abiotic stress-treated *Phaseolus vulgaris* and *Glycine max*. Screening of the library led to the selection of a new GST variant. The new enzyme was characterized by kinetic analysis and X-ray crystallography. The results demonstrated that random recombination of fragments from homologous GSTUs from different plants can give rise to new functionally synthetic GST enzyme.

## Results and discussion

### Analysis of the catalytic diversity of gstome from *P. vulgaris* and *G. max* under control and abiotic stress treatments

Transcriptomics and genomics projects have showed that plants have multiple genes coding for GSTs (Nianiou-Obeidat et al., 2017; Han et al., 2018). For example, in the *Glycine max* var. Williams 82 genome, 101 gene loci encode putative GSTs (Liu et al., 2015). The analysis of *P. vulgaris* trascriptomic and genomic data (available at https://phytozome.jgi.doe.gov) reveal the presence of at least 52 transcripts that encode putative GSTs (unpublished results). Plant GSTs are inducible enzymes that respond to biotic and abiotic stresses (Chronopoulou et al., 2012a; Csiszár et al., 2014; Pégeot et al., 2014; Han et al., 2018). In the present study, the induction of total GST activity in *P. vulgaris* and *G. max* tissues was evaluated in response to different chemical and physical stress agents to expand the repertoire of differently expressed GST isoenzymes with diverse catalytic and functional properties.

**Figure 1:**
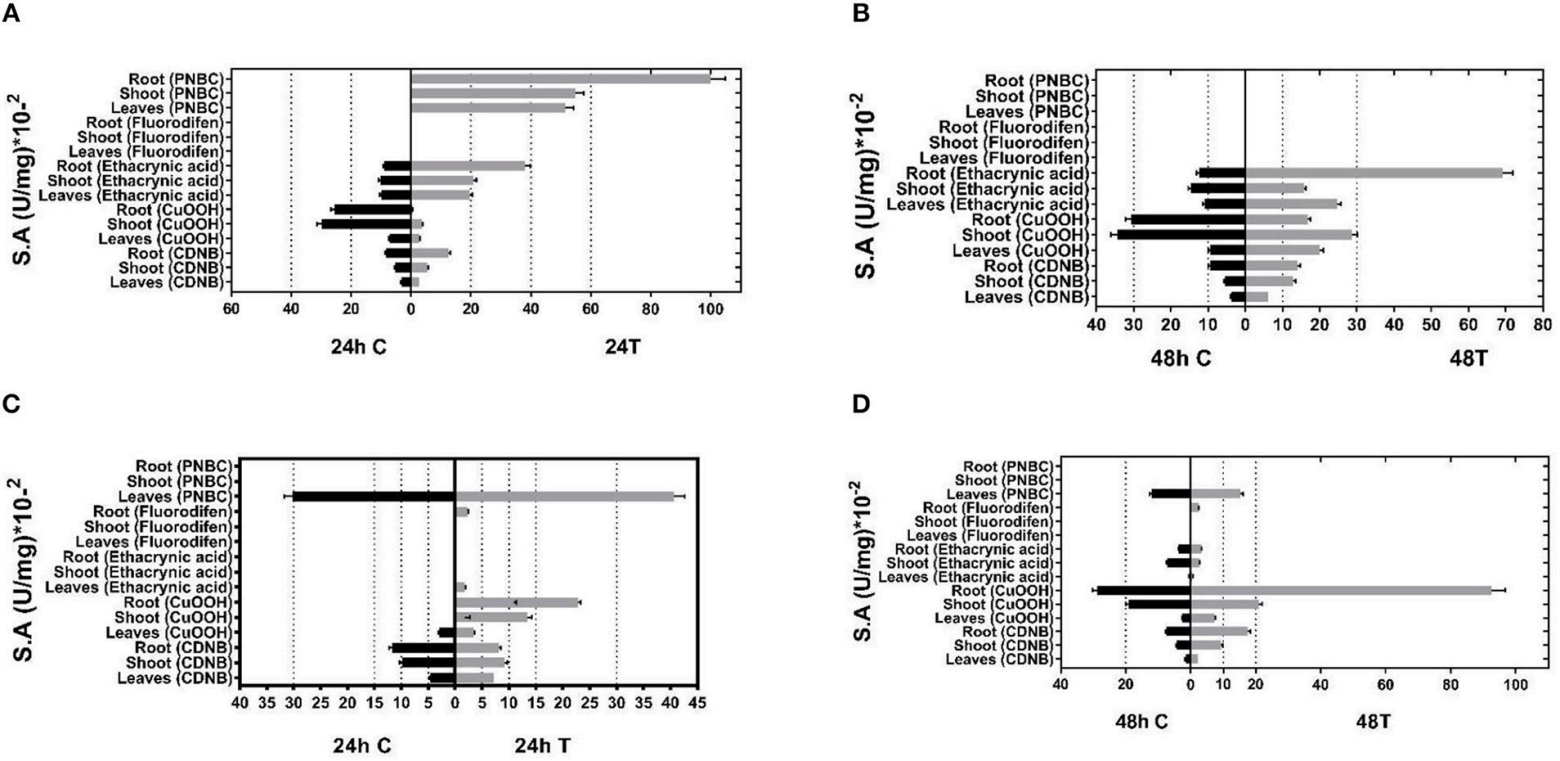
GST-specific activities of extracts from tissues (leaves, shoots, roots) of control and stressed plants after 24 and 48 h treatment for (**A**,**B**, respectively) and Phaseolus vulgaris (**C**,**D**, respectively) total activity was measured using CDNB, CuOOH, ethacrynic acid, fluorodifen, and NBD-Cl as substrates. Results represent the means of triplicate determinations, with variation less than 5% in all cases.

Given the inducible expression of GSTs under different abiotic stress conditions, young *P. vulgaris* and *G. max* plants were exposed to different abiotic stressors, such as a mixture of different herbicides (atrazine, alachlor, fluazifop-p-butyl), heavy metals (nickel, zinc, and chromium) as well as heat-shock (37°C). The purpose of these combined stress treatments was to invoke the expression of GST activities that are induced only following exposure to abiotic stresses (Kissoudis et al., 2015). Following the treatments, plants were harvested, homogenized, and crude extracts were assayed for GST activities using spectrophotometric assays and a range of different model substrates. Total GST activity was extracted from different plant tissues (leaf, root, and shoot) of both control plants and treated plants and measured using five different substrates: 1-chloro-2,4-dinitrobenzene (CDNB), cumene hydroperoxide (CuOOH), the herbicide fluorodifen, ethacrynic acid, and p-nitrobenzyl chloride (pNBC). The choice of these substrates was based on different chemistries involved in catalytic reactions (e.g., nucleophile substitution, addition, hydroperoxidation) to expand the possibilities of obtaining a broad range of catalytic functionalities. Prior experience has demonstrated that a high proportion of functional GSTs can be obtained by this approach (Chronopoulou et al., 2012a; Li et al., 2017a,b). The results (Figure 1) showed that the application of multiple stress conditions resulted in a large increase in total GST activity. For example, using CDNB as a substrate, a 1.4–2.3-fold increase was observed in different tissues, compared to the control plants in the 48 h treatment. CuOOH and ethacrynic acid produced a 1.1–3.2-fold and 1.1–5.6-fold increase in total GST activity, respectively. The results indicated that following abiotic stress treatment different GST isoenzymes were upregulated in different *P. vulgaris* and *G. max* tissues, suggesting an increased diversity in catalytic activities.

### Shuffling of cDNAs encoding GSTs from *P. vulgaris* and *G. max* and selection of a new variant

**Figure 2:**
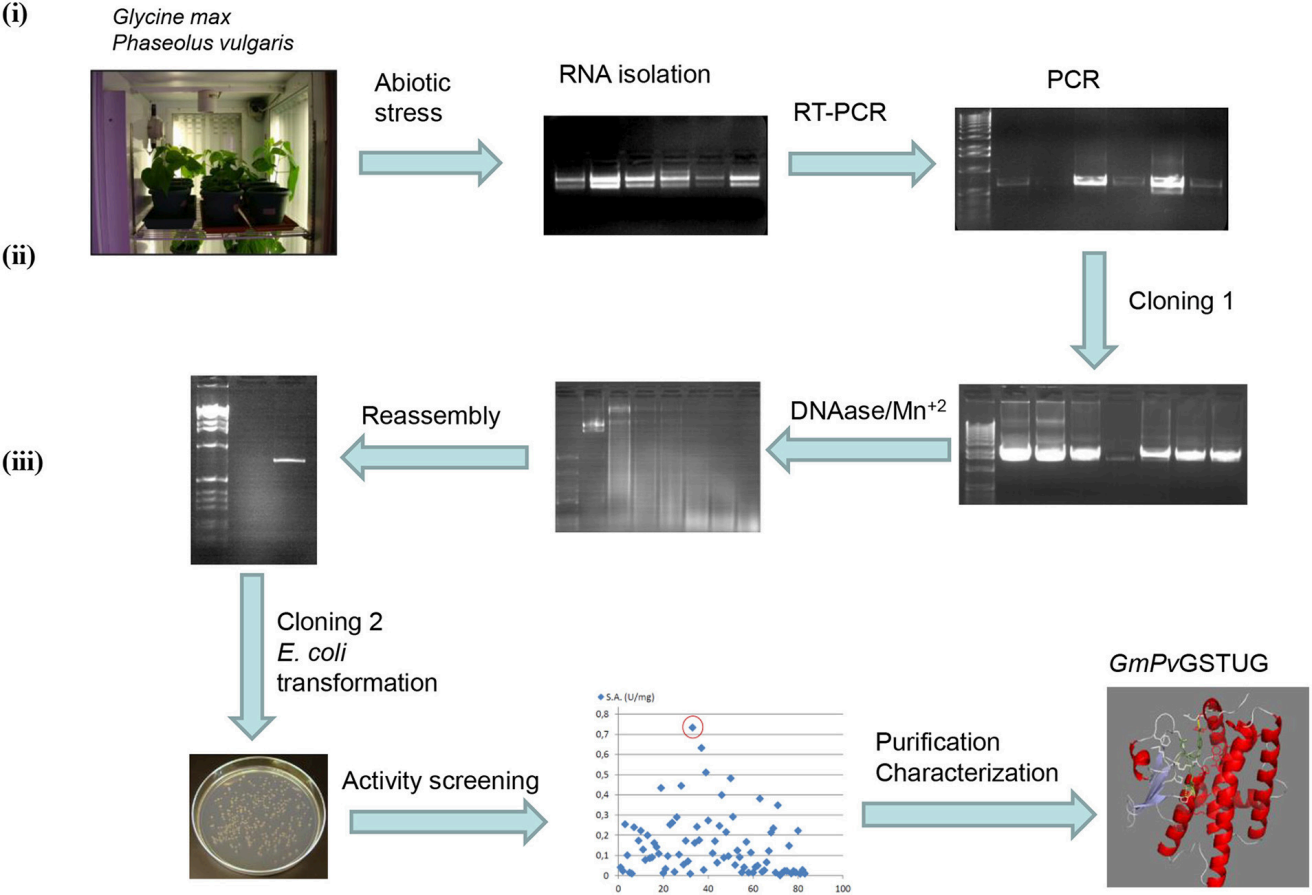
Schematic diagram of the experimental approach used for the generation of the synthetic GST gene and its corresponding enzyme *PvGm*GSTUG (i) Abiotic stress treatments of *Phaseolus vulgaris* and *Glycine max* plants lead to induction of total GST activity and allowed the creation of a GST-enriched cDNA library using degenerated GST-specific primers and reverse transcription-PCR; (ii) The GST-enriched library was further diversified employing directed evolution through DNA shuffling; (iii) Activity screening of the evolved library led to the isolation of a novel *tau* class GST enzyme (*PvGm*GST), which was purified and characterized.

**Figure 3:**
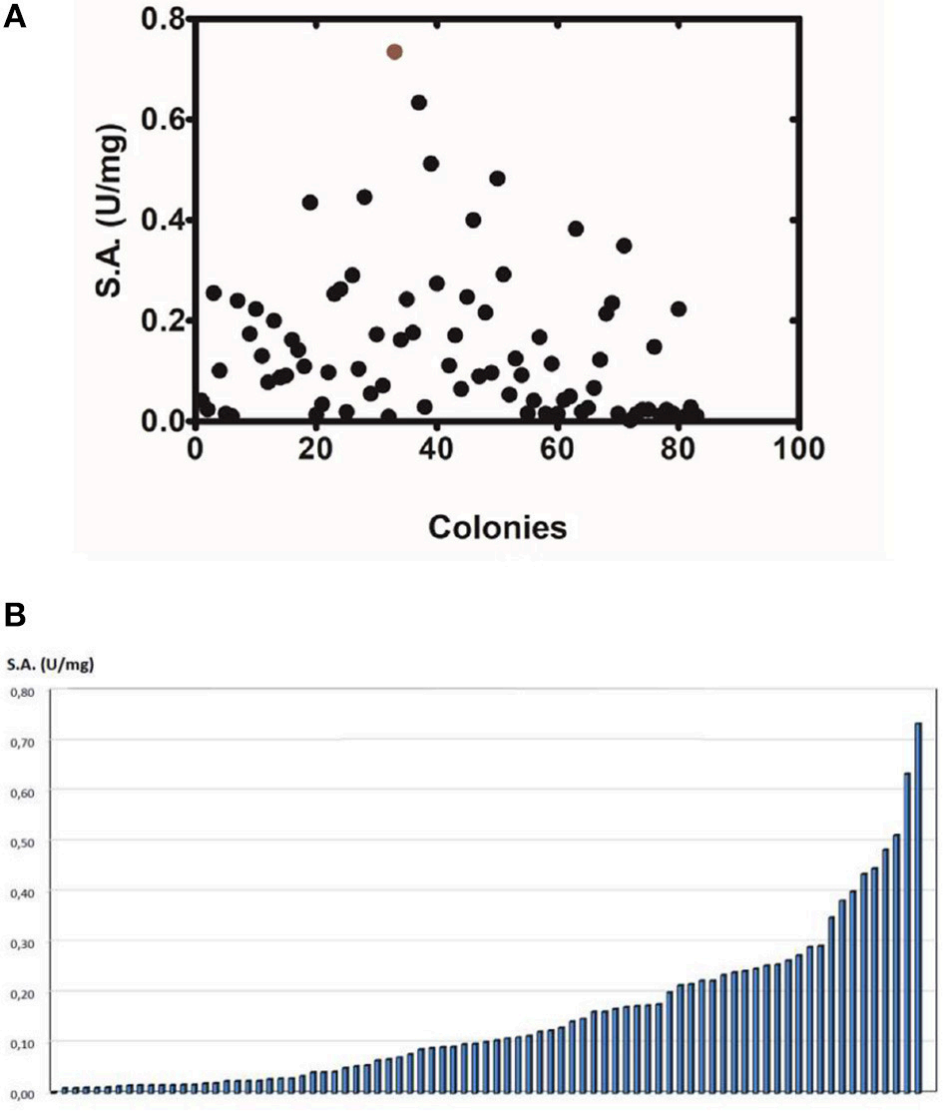
Activity screening **(A)**. Activity screening of different colonies obtained following DNA shuffling. The graph depicts only the colonies with detectable activity toward the substrate system CDNB/GSH. **(B)** Distribution of the specific activity of different colonies.

**Figure 4:**
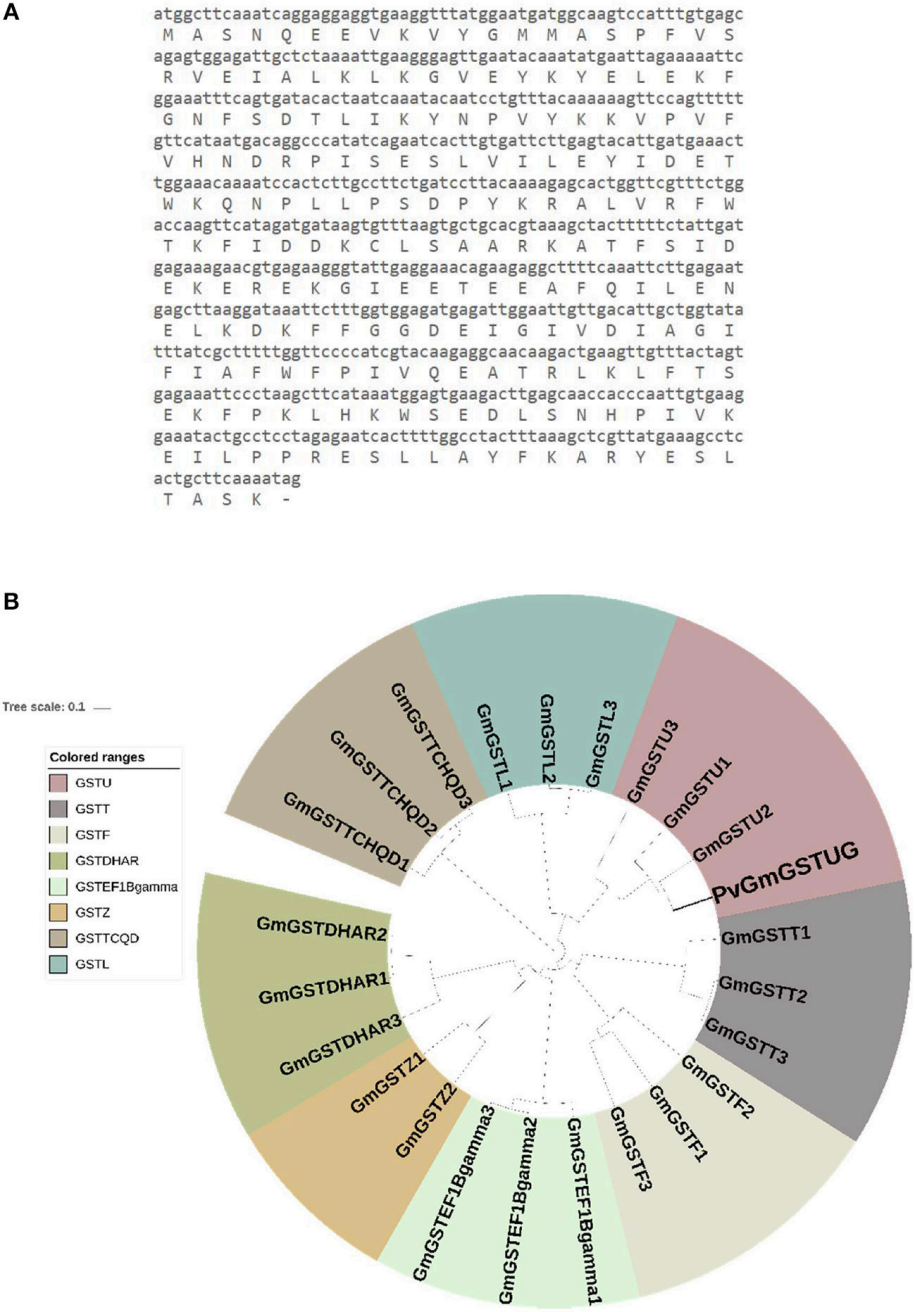
Sequence and phylogenetic analysis **(A)**. Nucleotide and amino acid sequence of the *PvGm*GSTUG **(B)**. Phylogenetic analysis of *PvGm*GSTUG with representative members from all classes of the *Glycine max* GST family. Sequences were aligned with the CLUSTAL Omega sequence alignment program (Sievers et al., 2011) and the phylogenetic tree was constructed using Geneious 9.1.2 software (http://www.geneious.com; Kearse et al., 2012) with the UPGMA tree building method and iTOL v1.0 software (Ciccarelli et al., 2006). Various classes can be distinguished: *Phi* (GSTF), *Tau* (GSTU), *Lambda* (GSTL), *Theta* (GSTT), *Dehydroascorbate reductase* (DHAR), *Elongation factor 1B*γ (EF1Bγ), *Zeta* (GSTZ), and *Tetrachloro-hydroquinone dehalogenase* (TCHQD). The accession numbers of Glycine max GSTs that were used for this phylogenetic tree are: *Phi* class: *Gm*GSTF1 (AJE59615.1), *Gm*GSTF2 (AJE59616.1), *Gm*GSTF3 (AJE59618.1), *Tau* class: *Gm*GSTU1 (AJE59646.1), *Gm*GSTU2 (AJE59647.1), *Gm*GSTU3 (AJE59651.1), *Lambda* class: *Gm*GSTL1 (AJE59633.1), *Gm*GSTL2 (AJE59634.1), *Gm*GSTL3 (AJE59635.1), *Theta* class: *Gm*GSTT1 (AJE59641.1), *Gm*GSTT2 (AJE59642.1), *Gm*GSTT3 (AJE59643.1), *DHAR* class: *Gm*GSTDHAR1 (AJE59631.1), *Gm*GSTDHAR2 (AJE59630.1), *Gm*GSTDHAR3 (AJE59629.1), *EF1Bgamma* class: *Gm*GSTEF1Bgamma1 (AJE59625.1),*Gm*GSTEF1Bgamma2 (AJE59626.1), *Gm*GSTEF1Bgamma3 (AJE59627.1) *Zeta* class: *Gm*GSTZ2 (AJE59689.1), *Gm*GSTZ1 (AJE59691.1) *TCHQD* class: *Gm*GSTTCHQD1 (AJE59638.1), *Gm*GSTTCHQD2 (AJE59639.1), and *Gm*GSTTCHQD3 (AJE59640.1).

**Figure 5:**
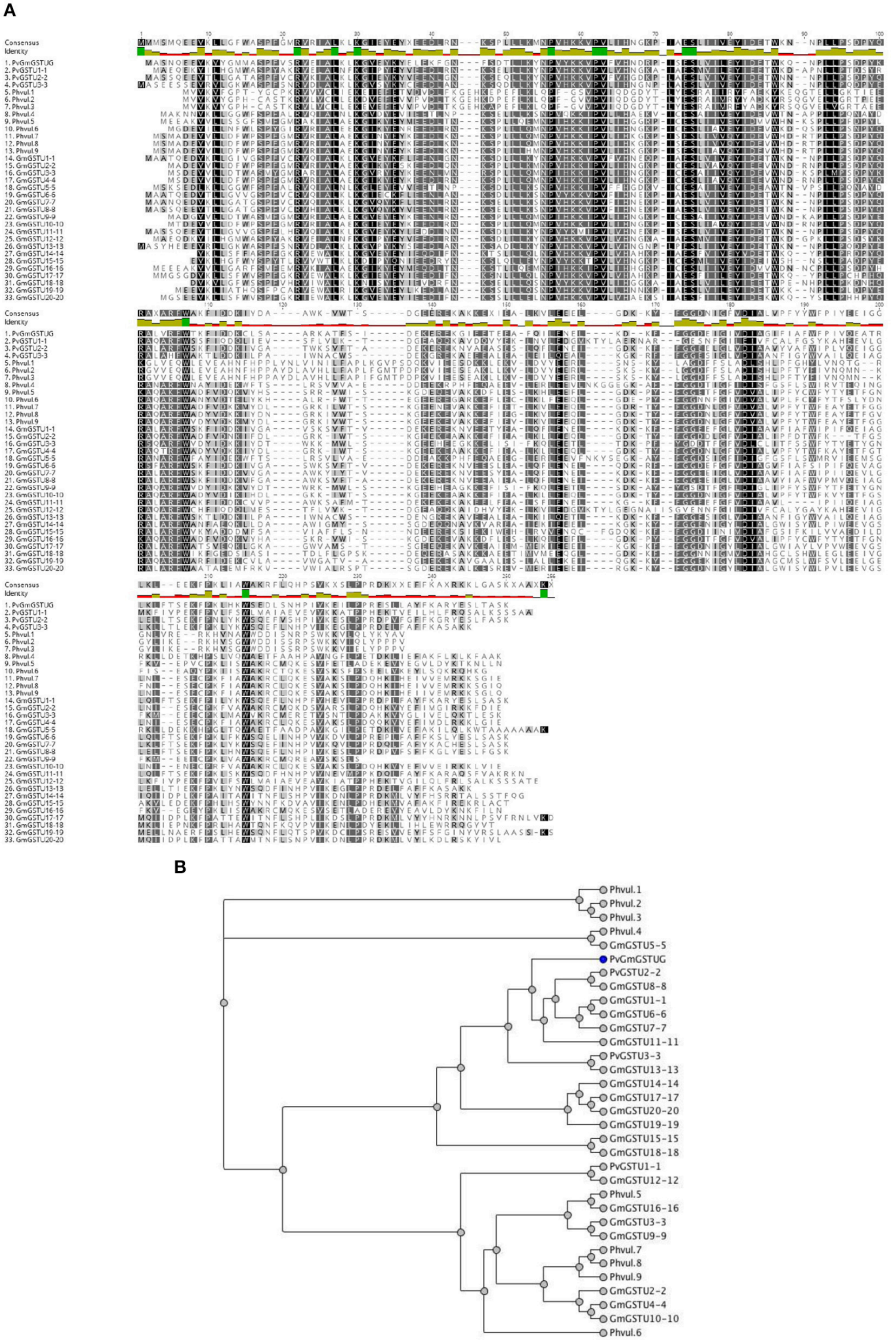
Sequence and phylogenetic analysis **(A)**. Amino acid sequence alignments of *PvGm*GSTUG with the tau class GSTs from *Glycine max* and *Phaseolus vulgaris* **(B)**. Phylogenetic analysis of *GmPv*GSTUG with the tau class GSTs from *Glycine max* and *Phaseolus vulgaris*. Phylogenetic tree was constructed by the neighbor joining method using Geneious v9.1.2 software (Kearse et al., 2012) after alignment of the protein sequences using the Clustal Omega sequence alignment program (Sievers et al., 2011). The figures were created using Geneious v9.1.2 software (Kearse et al., 2012). Conserved areas are shown shaded: 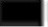 100% identity, 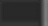 80–100% identity, 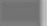 60–80% identity, < 60% identity. The accession numbers and gene codes of the GST sequences that were used were: PvGSTU1-1 (AEX38000.1); PvGSTU2-2 (AEX38001.1); PvGSTU3-3 (NP_171792); Phvul.1 (006G023500.1|PACid:27165305); Phvul.2 (008G195500.1|PACid:27155547); Phvul.3 (008G195600.1|PACid:27155113); Phvul.4 (002G080200.1|PACid:27169916); Phvul.5 (005G053300.1 PACid:27149482); Phvul.6 (005G053200.1|PACid:27149239); Phvul.7 (005G054000.1|PACid:27150418); Phvul.8 (code 005G054100.1|PACid:27148744); and Phvul.9 (005G054200.1|PACid:27149131). The accession numbers of *Glycine max* GST sequences that were used were: *Gm*GSTU1-1, AAA33973; *Gm*GSTU2-2, CAA71784; *Gm*GSTU3-3, CAA48717; *Gm*GSTU4-4, AAC18566; *Gm*GSTU5-5, AAG34795; *Gm*GSTU6-6, AAG34796; *Gm*GSTU7-7, AAG34797; *Gm*GSTU8-8, AAG34798; *Gm*GSTU9-9, AAG34799; *Gm*GSTU10-10, AAG34800; *Gm*GSTU11-11, AAG34801; *Gm*GSTU12-12, AAG34802; *Gm*GSTU13-13, AAG34803; *Gm*GSTU14-14, AAG34804; *Gm*GSTU15-15, AAG34805; *Gm*GSTU16-16, AAG34806; *Gm*GSTU17-17, AAG34807; *Gm*GSTU18-18, AAG34808; *Gm*GSTU19-19, AAG34809; and *Gm*GSTU20-20, AAG34810.

The method of DNA shuffling is an effective strategy for generating genetic diversity and for identifying protein variants with improved or altered functional or structural properties. The DNA shuffling protocol consists of the following steps: (i) selection and preparation of genes to be shuffled, (ii) digestion of the selected genes with DNase I for generation of a mixture of DNA fragments (size 50–100 bp), (iii) reassembly of DNA fragments with PCR without primers, and (iv) amplification of reassembled products by a conventional PCR. During the PCR reactions, point mutations may be generated. Abiotic stress treatment of *P. vulgaris* and *G. max* makes them perfect starting materials for producing a cDNA library enriched with GSTs (Figure 2). Thus, RNA from stressed tissues (leaf, root, and shoot treated for 48 h) was reverse transcribed and the GST genes were amplified using PCRs and degenerate primers. The PCR amplicons of putative GST genes were cloned and the resulted recombinant plasmids were isolated, mixed, and used for *in vitro* recombination by DNA shuffling (Zhao and Arnold, 1997; Axarli et al., 2016, 2017). Following *in vitro* recombination, a single PCR product was cloned into the pEXP5-CT/TOPO®TA plasmid. Different colonies (180 in total) were screened for GST activity. Approximately 46% of the picked colonies exhibited GST activity, suggesting that the recombination produced a library with high proportion of catalytically active GSTs (Figure 3). Interestingly, the mean specific activity was 0.17 U/mg and 52% of the active colonies displayed specific activity higher than 0.1 U/mg. The GST variant that displayed the highest activity was selected for further characterization. This clone was sequenced (Figure 4A) and revealed a 672 bp open reading frame encoding a protein of 224 amino acid residues with a molecular mass of 26,088.08 Da and a theoretical pI of 5.80. BLAST searches showed that both its nucleotide (BLASTN) as well as amino acid sequences (BLASTP) were novel and absent from all public databases (Tables 1, 2; Supplementary Figures 1, 2). The phylogenetic relationship of this new enzyme with other GSTs from all known classes was investigated by the construction of a phylogenetic tree that was generated by multiple amino acid sequence alignment (Figure 4B). The alignment was created using representative members of all classes of the *Glycine max* GST family (*Gm*GSTs) (McGonigle et al., 2000; Liu et al., 2015). The enzyme that resulted from DNA shuffling, which was denoted as *PvGm*GSTUG in accordance with the nomenclature proposed by Edwards et al. (2000), clustered together with the tau class GSTs. Of note, as evident from the data provided in Tables 1, 2, *PvGm*GSTUG displayed the highest homology (87 and 86 % homology at the nucleotide and amino acid level, respectively,) with a GST from *Medicago truncatula* (nucleotide and amino acid accession codes XM_003623148.2 and XP_003623196.1, respectively), rather than the GSTs from *Phaseolus vulgaris* and *Glycine max*, in excellent agreement with their evolution history. This important observation further supports the evolution theory of legume plants (Cronk et al., 2006). Amino acid sequence alignments and phylogenetic analysis of *PvGm*GSTUG with the *tau* class GSTs from *G. max* and *P. vulgaris* revealed that the *PvGm*GSTUG displayed higher identity with the *Pv*GSTU2-2 and *Gm*GSTU8-8 isoenzymes and, from the evolutionary point of view, formed a separate clade (Figure 5). Although the accurate prediction of the parent sequences was impossible, we can nevertheless speculate that most of the *PvGm*GSTUG sequence was derived from *Pv*GSTU2-2 (Chronopoulou et al., 2012a) and *Gm*GSTU8-8 (Pouliou et al., 2017).

**Table 1:** Percent amino acid identity matrix of *PvGm*GSTUG with the first 12 sequences identified in the BLASTP search.

	***PvGm*GSTUG**	***Mt*GS1**	***Mt*GST2**	***Mt*GST3**	***Ts*Pr1**	***Ts*Pr2**	***Ca*GST1**	***Ca*GST2**	***Mt*GST4**	***Mt*GST5**	***Ts*Pr3**	***Mt*GST6**
1:*PvGm*GSTUG	**100.00**	85.71	77.68	77.68	77.23	75.00	74.55	72.77	73.66	76.26	71.82	71.43
2:*Mt*GST1	85.71	**100.00**	79.91	78.12	77.23	75.00	78.57	74.55	75.00	78.54	72.73	74.55
3:*Mt*GST2	77.68	79.91	**100.00**	79.02	80.36	77.68	74.55	74.55	77.23	80.37	76.36	75.45
4:*Mt*GST3	77.68	78.12	79.02	**100.00**	74.55	73.66	74.11	75.45	89.29	81.28	80.00	86.61
5:*Ts*Pr1	77.23	77.23	80.36	74.55	**100.00**	87.95	75.89	73.66	74.11	79.91	76.82	70.54
6:*Ts*Pr2	75.00	75.00	77.68	73.66	87.95	**100.00**	70.54	69.64	72.32	77.17	72.73	69.20
7:*Ca*GST1	74.55	78.57	74.55	74.11	75.89	70.54	**100.00**	79.46	72.32	75.34	73.64	71.43
8:*Ca*GST2	72.77	74.55	74.55	75.45	73.66	69.64	79.46	**100.00**	74.11	75.80	72.27	72.77
9:*Mt*GST4	73.66	75.00	77.23	89.29	74.11	72.32	72.32	74.11	**100.00**	80.37	77.73	87.50
10:*Mt*GST5	76.26	78.54	80.37	81.28	79.91	77.17	75.34	75.80	80.37	**100.00**	79.07	76.26
11:*Ts*Pr3	71.82	72.73	76.36	80.00	76.82	72.73	73.64	72.27	77.73	79.07	**100.00**	74.55
12:*Mt*GST6	71.43	74.55	75.45	86.61	70.54	69.20	71.43	72.77	87.50	76.26	74.55	**100.00**

*For the analysis, the amino acid sequence of PvGmGSTUG was used in the query. Percent identity matrix was calculated with the CLUSTAL Omega sequence alignment program (Sievers et al., 2011). The accession numbers of the GST sequences that resulted from the searches were: MtGST.1, (Medicago truncatula GST, XP_003623196.1); MtGST.2, (Medicago truncatula GST, XP_003623195.1); MtGST.3, (Medicago truncatula GST, XP_003623174.1); CaGST1, (Cicer arietinum GST3, ALZ41813.1); CaGST2, (Cicer arietinum GST, XP_004492376.1); MtGST4, (Medicago truncatula GST, XP_013449023.1); MtGST5, (Medicago truncatula GST, XP_003623168.1); MtGST6, (Medicago truncatula GST, XP_003623173.1); LaGST, (Lupinus angustifolius GST, XP_019459310.1); MtGST7, (Medicago truncatula GST, XP_003623171.2); GmGST1, (Glycine max GST, NP_001238439.1); CcGST, (Cajanus cajan GST, XP_020206357.1); GmGST2, (Glycine max GST, NP_001304556.1); GsGST1, (Glycine soja, KHN05112.1); and GsGST2, (Glycine soja, KHN06986.1). The GST isoenzymes and 100% identity are in bold*.

**Table 2:** Percent nucleotide identity matrix of *PvGm*GSTUG with the first 12 sequences identified in the BLASTP search.

	***PvGm*GSTUG**	***Mt*GST1**	***Ca*GST1**	***Mt*GST2**	***Mt*GST3**	***Ca*GST2**	***Ca*GST3**	***Mt*GST4**	***Mt*GST5**	***Mt*Pr1**	***Ai*GST1**	***Ai*GST2**
*1.PvGm*GSTUG	**100.00**	87.26	82.37	82.22	81.97	75.85	74.66	75.23	74.21	74.06	72.35	71.45
*2.Mt*GST1	87.26	**100.00**	82.81	83.26	85.91	74.67	75.87	75.99	73.91	73.76	73.24	72.80
*3.Ca*GST1	82.37	82.81	**100.00**	83.70	83.48	74.22	74.96	74.31	73.00	72.85	70.55	70.10
*4.Mt*GST2	82.22	83.26	83.70	**100.00**	86.52	75.85	73.76	74.01	72.55	72.40	73.09	72.65
*5.Mt*GST3	81.97	85.91	83.48	86.52	**100.00**	73.03	74.92	75.19	74.01	73.85	70.95	70.49
*6.Ca*GST2	75.85	74.67	74.22	75.85	73.03	**100.00**	76.47	75.99	74.81	74.66	74.29	73.39
*7.Ca*GST3	74.66	75.87	74.96	73.76	74.92	76.47	**100.00**	85.08	84.68	84.53	72.23	72.23
*8.Mt*GST4	75.23	75.99	74.31	74.01	75.19	75.99	85.08	**100.00**	87.98	87.82	71.43	71.27
*9.Mt*GST5	74.21	73.91	73.00	72.55	74.01	74.81	84.68	87.98	**100.00**	99.85	71.32	71.32
*10.Mt*Pr1	74.06	73.76	72.85	72.40	73.85	74.66	84.53	87.82	99.85	**100.00**	71.17	71.17
*11.Ai*GST1	72.35	73.24	70.55	73.09	70.95	74.29	72.23	71.43	71.32	71.17	**100.00**	97.32
*12.Ai*GST2	71.45	72.80	70.10	72.65	70.49	73.39	72.23	71.27	71.32	71.17	97.32	**100.00**

*For the analysis, the nucleotide sequence of PvGmGSTUG was used in the query. Percent identity matrix was calculated with the CLUSTAL omega sequence alignment program (Sievers et al. 2011). The accession numbers of the sequences are: MtGST1, (Medicago truncatula GST, XM_003623148.2); CaGST1, (Cicer arietinum GST, XM_004492319.2); MtGST2, (Medicago truncatula GST, XM_003623126.2); MtGST3, (Medicago truncatula GST, XM_003623120.2); CaGST2, (Cicer arietinum GST: KT336759.1); CaGST3, (Cicer arietinum GST, XM_012713550.1); MtGST4, (Medicago truncatula GST, XM_003623159.1); MtGST5, (Medicago truncatula GST 5, XM_003623156.2); MtPr1, (Medicago truncatula GST:BT053471.1); AiGST1 (Arachis ipaensis GST:XM_016342954.2); and AiGST2 (Arachis ipaensis GST: XM_016333558.2). The GST isoenzymes and 100% identity are in bold*.

### Substrate specificity and kinetic analysis of *PvGm*GSTUG enzyme

**Figure 6:**
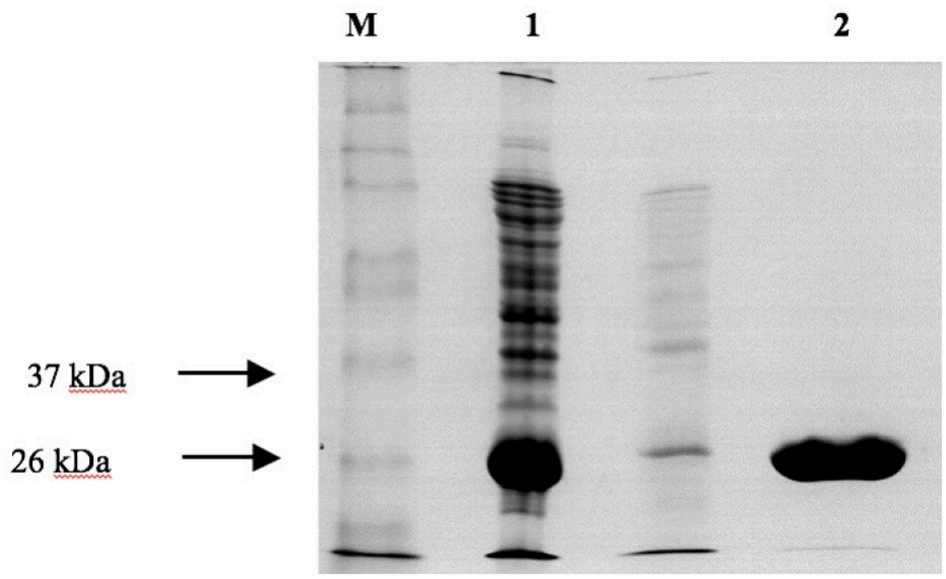
SDS-PAGE analysis of the purification of recombinant *PvGm*GSTUG by affinity chromatography on S-hexyl-GSH-agarose M denotes the molecular mass markers. Lane 1 contains recombinant *E. coli* BL21 (DE3) crude extract after induction with 1 mM IPTG. Lane 2 contains *PvGm*GSTUG eluted fractions from the S-hexyl-GSH-Sepharose. Elution was achieved with 10 mM GSH.

Recombinant *PvGm*GSTUG was purified to homogeneity by affinity chromatography on S-hexyl-GSH-agarose adsorbent (Figure 6). The substrate specificity of *PvGm*GSTUG was evaluated using a broad range of substrates. The results (Table 3) showed that *PvGm*GSTs could catalyze a broad range of reactions. Several halogenated aromatic compounds were acceptable substrates. They included CDNB and its analogs: 1-bromo-2,4-dinitrobenzene (BDNB), 1-iodo-2,4-dinitrobenzene (IDNB), and 4-chloro-7-nitrobenzofurazan. *PvGm*GSTUG was also examined for GST-dependent peroxidase activity (GPOX) using CuOOH, tert-butyl hydroperoxide, and benzoyl peroxide as substrates. Among all the peroxides tested, CuOOH and lauroyl peroxide were the best substrates. *PvGm*GSTUG also catalyzed the conjugation of GSH with isothiocyanates. *PvGm*GST, displayed high catalytic activity toward the aliphatic allyl-isothiocyanate, compared to the aromatic phenethyl-isothiocyanate.

**Table 3:** Substrate specificity for purified recombinant *PvGm*GSTUG.

**Substrate**	**Structure**	**Specific activity (U/mg)**
1-Chloro-2,4-dinitrobenzene	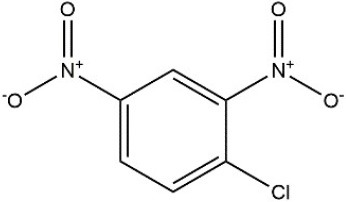	14.6
1-Bromo-2,4-dinitrobenzene	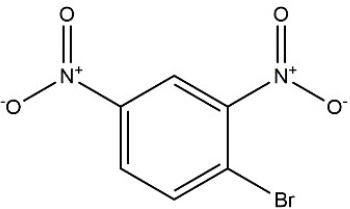	6.9
1-Fluoro-2,4-dinitrobenzene	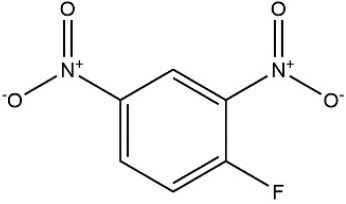	ND
1-Iodo-2,4-dinitrobenzene	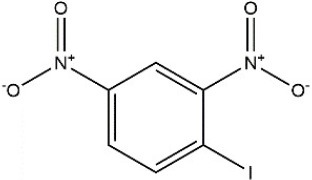	0.8
p-Nitrobenzyl chloride	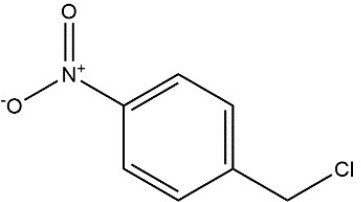	ND
4-Chloro-7-nitrobenzofurazan	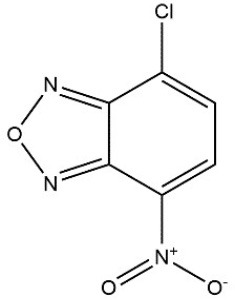	4.5
Cumene hydroperoxide	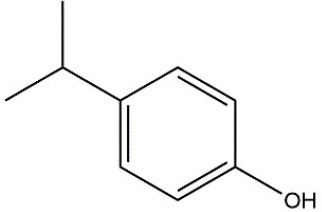	6.64
*t*-Butyl hydroperoxide	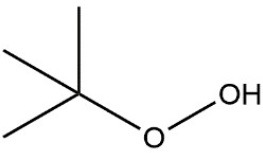	0.5
Benzoyl peroxide	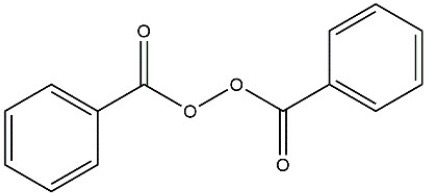	ND
Trans-2-Nonenal	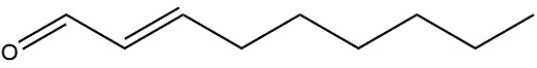	0.07
Trans-4-Phenyl-3-buten-2-one	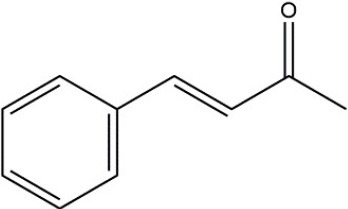	ND
Ethacrynic acid	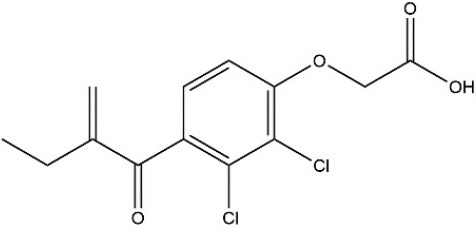	1.2
Fluorodifen	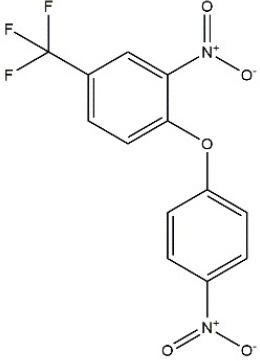	ND
Allylisothiocyanate	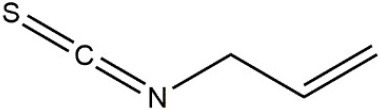	7.3
Phenethylisothiocyanate	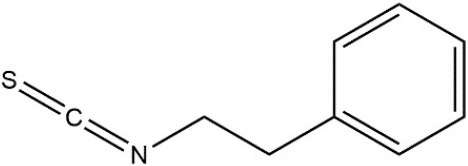	1.6
2-Hydroxyethyl disulfide (2-2-dithiodiethanol)	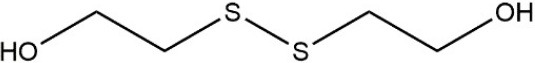	ND
Dehydroascorbate	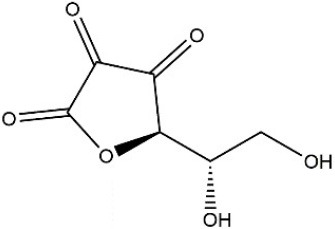	ND
Bromosulfophthalein	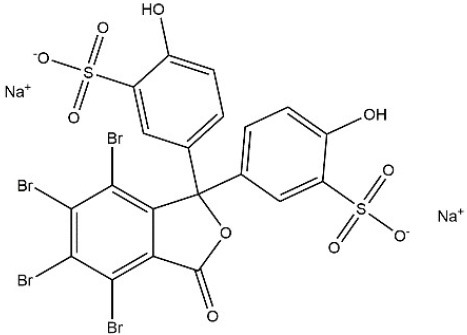	ND

ND: Non-detectable enzyme activity

*Results represent the means of triplicate determinations, with variation less than 5% in all cases*.

**Figure 7:**
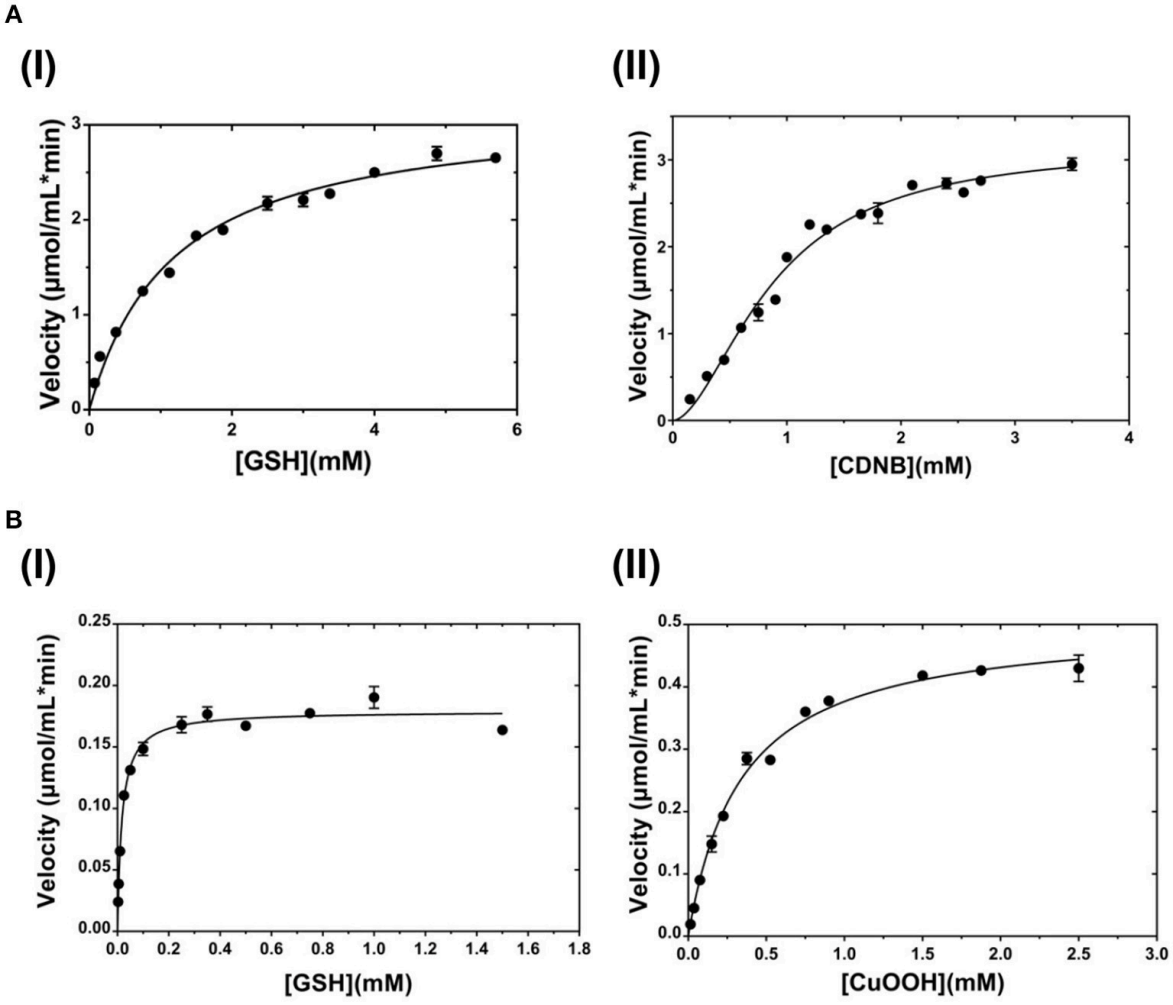
Steady-state kinetic analysis **(A)**. Steady-state kinetic analysis of *PvGm*STUG using GSH as a variable substrate (I) and CDNB at a fixed concentration. Steady-state kinetic analysis of *PvGm*STUG using the CDNB as a variable substrate (II) and GSH at a fixed concentration **(B)**. Steady-state kinetic analysis of *PvGm*STUG using GSH as a variable substrate (I) and CuOOH at a fixed concentration. Steady-state kinetic analysis of *PvGm*STUG using the CuOOH as a variable substrate (II) and GSH at a fixed concentration. Experiments were performed in triplicate.

The dependence of catalytic activity of *PvGm*GSTUG enzyme was investigated using steady-state kinetic analysis. The analysis was performed by employing two different model reaction systems: the GSH/CDNB and the GSH/CuOOH (Figure 7). The GSH/CDNB is a typical SN2 nucleophilic substitution reaction whereas the GSH/CuOOH reaction is an oxidative reaction (e.g., hydroperoxidase activity). The results are summarized in Table 4. *PvGm*GSTUG obeyed normal Michaelis-Menten kinetics when GSH was used as a variable substrate in both types of reactions. The unusual low K_m_ value (K_m_ 17 ± 1 μM) obtained for GSH in its reaction with CuOOH suggested that the enzyme is able to perform efficient catalysis under physiological conditions where the concentration of GSH is reduced, as for example under oxidative stress (Skopelitou et al., 2017).

**Table 4:** Steady-state kinetic parameters of *PvGm*GSTUG for the CDNB/GSH substrate system (A) and for the CuOOH/GSH substrate system (B).

**Substrate system**	**K_m_ (mM) (GSH)**	**S_0.5_ (mM) (CDNB)**	**k_cat_ (min^−1^) (GSH)**	**n_H_ (CDNB)**	**k_cat_/K_m_ (mM^−1^ min^−1^) (GSH)**	**k_cat_/S_0.5_ (mM^−1^ min^−1^) (CDNB)**
**A**						
**CDNB/GSH**	1.17 ± 0.09	0.88 ± 0.05	194.1 ± 4.85	1.77 ± 0.14	165.9 ± 0.14	217.5
**Substrate system**	**K**_m_ **(mM) (GSH)**	**K**_m_ **(mM) (CuOOH)**	**k**_cat_ **(min**^−1^**) (GSH)**	**k**_cat_**/K**_m_ **(mM**^−1^ **min**^−1^**) (GSH)**	**k**_cat_**/K**_m_ **(m**^−1^ **min**^-1^**) (CuOOH)**
**B**					
**CuOOH/GSH**	0.017 ± 0.001	0.34 ± 0.02	29.61 ± 0.32	1,741.5 ± 127.9	87.1 ± 3.08

When CDNB was used as the variable substrate, the enzyme showed cooperative allosteric kinetics. A Hill coefficient (n_H_) of 1.8 ± 0.1 was measured with CDNB. Previous studies have established that in several *tau* class GSTs, although the H-site of neighboring subunits is remote, a reasonable communication between them exists. For example, in the case of a mutant form of *Gm*GST4-4 (Axarli et al., 2016) structural examination revealed that Lys104, which is located at the dimer interface, plays a key role in inter-subunit communication as well as in the cooperative allosteric kinetics observed with this enzyme.

### Thermal stability

**Figure 8:**
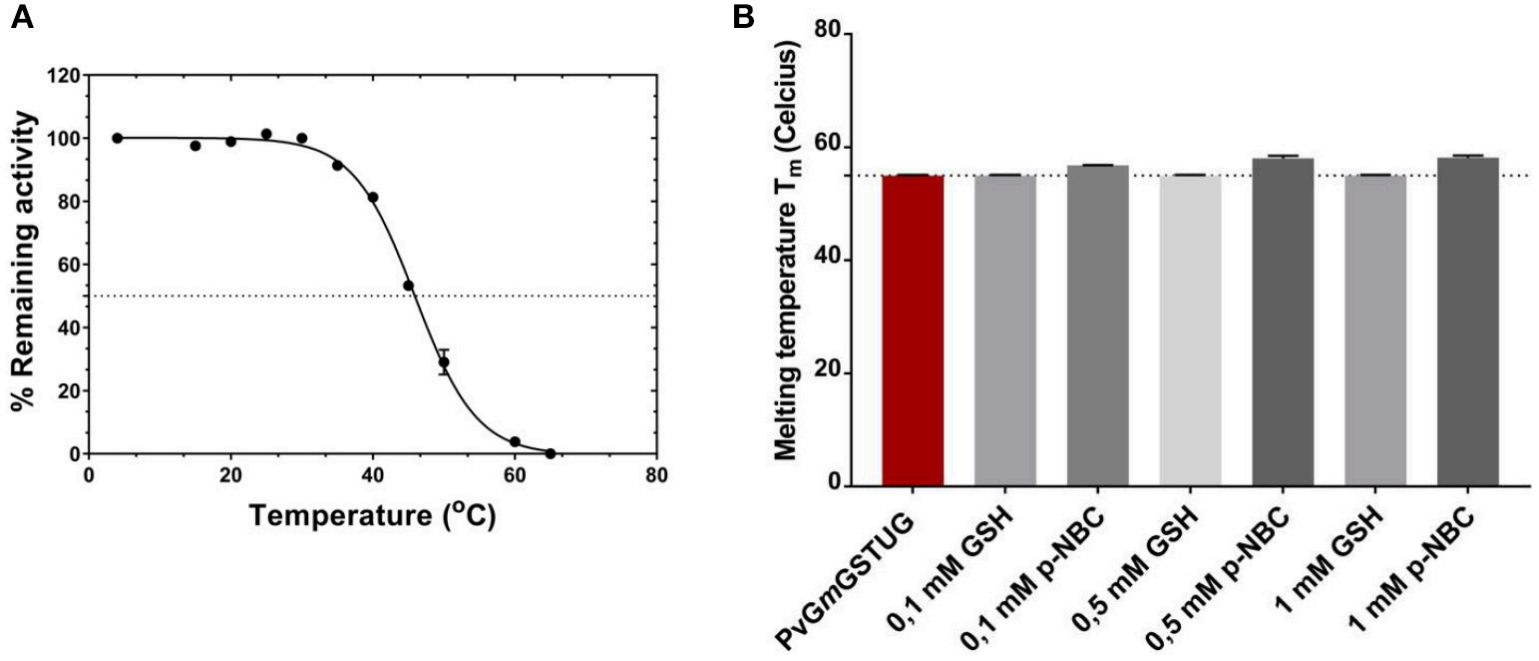
Thermal inactivation curves for *PvGm*GSTUG **(A)**. Thermal inactivation curves for *PvGm*GSTUG. The residual activities were measured after heat treatment at various temperatures (°C) for 5 min **(B)**. Histogram depicting melting temperature of the protein in the different conditions tested.

To evaluate whether the simultaneous shuffling of GST genes from different plants allowed the generation of structurally stable GST fold, thermal inactivation and unfolding measurements were achieved as illustrated in Figure 8A. The half-inactivation temperature (T_m_) was 45.9 ± 0.2°C, which lies within the expected range for mesophilic enzyme and is close to that determined for other native GST isoenzymes (Skopelitou et al., 2017; Perperopoulou et al., 2018). This suggests that the *PvGm*GSTUG structure displays normal stability and that no detrimental mutations or insertions were introduced during the shuffling of GST genes.

Differential scanning fluorometry (DSF) was also performed to assess the temperature-induced unfolding of the enzyme. DSF was carried out in the absence (Figure 8B) or presence of different concentrations of the substrate (GSH) and the reaction product [S-(p-nitrobenzyl)-GSH] (Figures 8C,D). The unfolding profile of the free enzyme as well as of the enzyme-GSH complex exhibited a single transition with a symmetric peak, with the maximum fluorescence intensity, corresponding to T_m_, at 55 ± 0.1°C (*n* = 4) (Supplementary Figures 3A-C). On the other hand, in the presence of S-(p-nitrobenzyl)-GSH, an increase of the protein Gibbs free energy of unfolding was observed, which usually is depicted as a T_m_ shift at higher temperatures (Supplementary Figure 3C) (Lea and Simeonov, 2012). This T_m_ shift suggested a more stable structure with a closed, compact conformation, compared to that of the free enzyme or the enzyme-GSH complex, an indication of an induced-fit mechanism of *PvGm*GSTUG catalysis (Axarli et al., 2009a; Figure 8B).

### Crystallographic analysis and structural characterization of *PvGm*GSTUG

**Figure 9:**
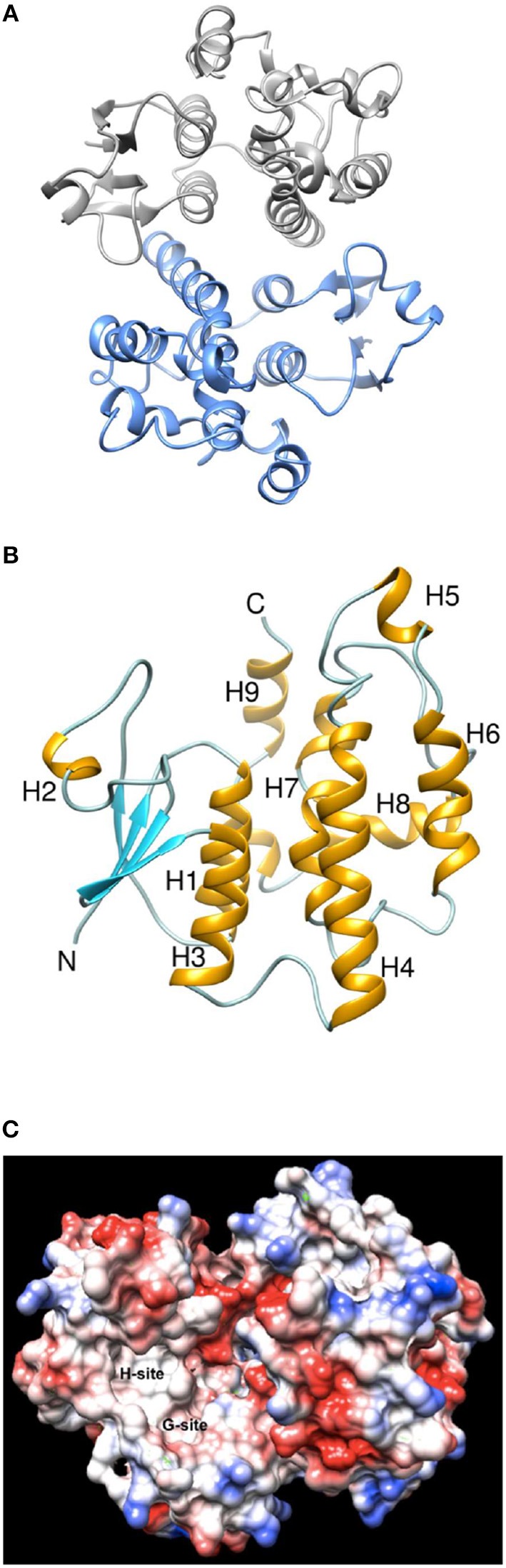
Ribbon representation and Coulombic surface analysis of the *PvGm*GSTUG dimer. **(A)** Ribbon representation of the *PvGm*GSTUG dimer. Each subunit is shown in a different color. The two-fold axis that relates the two subunits is perpendicular to the plane of the page. **(B)** Ribbon representation of *PvGm*GSTUG subunit. Helices and strands are shown in orange and cyan, respectively. The helices are labeled. The figures were using CHIMERA (Pettersen et al., 2004). **(C)** Coulombic surface analysis of *PvGm*GSTUG dimer. The analysis was carried out using UCSF Chimera (http://www.cgl.ucsf.edu/chimera). The Coulomb electrostatic surface shows regions of neutral (white), positive (blue), and negative (red) charge.

To better understand its properties, *PvGm*GSTUG was subjected to structural determination by X-ray crystallography (Figure 9). *PvGm*GSTUG was crystallized with two molecules in the crystallographic asymmetric unit that followed the typical dimer formation found in other GSTs (Axarli et al., 2009a; Pégeot et al., 2014; Skopelitou et al., 2017). The final structure (Table 5) displayed good geometry with 93.7% of the residues in the preferred and accepted regions of the Ramachandran plot and 6.3% in the disallowed regions. Residues 1–5 in both chains, and the fragments 214–224 (chain B), and 216–224 (chain A) were not included in the structure owing to high disorder. The root mean square deviation in bond length and angle was 0.010 Å and 1.52°, respectively. The analysis revealed that each monomer of *PvGm*GSTUG consists of two distinct domains: at the N-terminal region a small α/β thioredoxin-like domain with β*αβαββα* folding topology is formed. The topology is arranged in the order β2, β1, β3, and β4. At the C-terminal region a large helical domain is formed (Figure 8B). At the end of helix H3 a short linker (residues 79–91) begins that joins the N- and C-terminal domains.

**Table 5:** Data collection and refinement statistics.

**Beamline**	**ESRF ID23-1**
Wavelength (Å)	0.9730
Resolution range (Å)	50.0–3.5 (3.6–3.5)^#^
Space group	*P*4^3^
Cell parameters
a, b, c (Å) α = β = γ (°)	51, 51, 227.5 90
Total observations/unique	34,044/7132
Completeness	98.6 (99.0)
*R*_meas_	0.099 (1.38)
CC_1/2_	0.998 (0.656)
Reflections used in refinement (work/free)	6,380/709
*R*_work_/*R*_free_	0.29/0.34
Number of non-hydrogen atoms	3,431
RMS bonds (Å)	0.010
RMS angles (°)	1.52
Clashscore	14.4
B-factor (Å^2^)	80.5
PDB id	6GHF

# *Numbers in parenthesis refer to the outermost resolution shell*.

**Figure 10:**
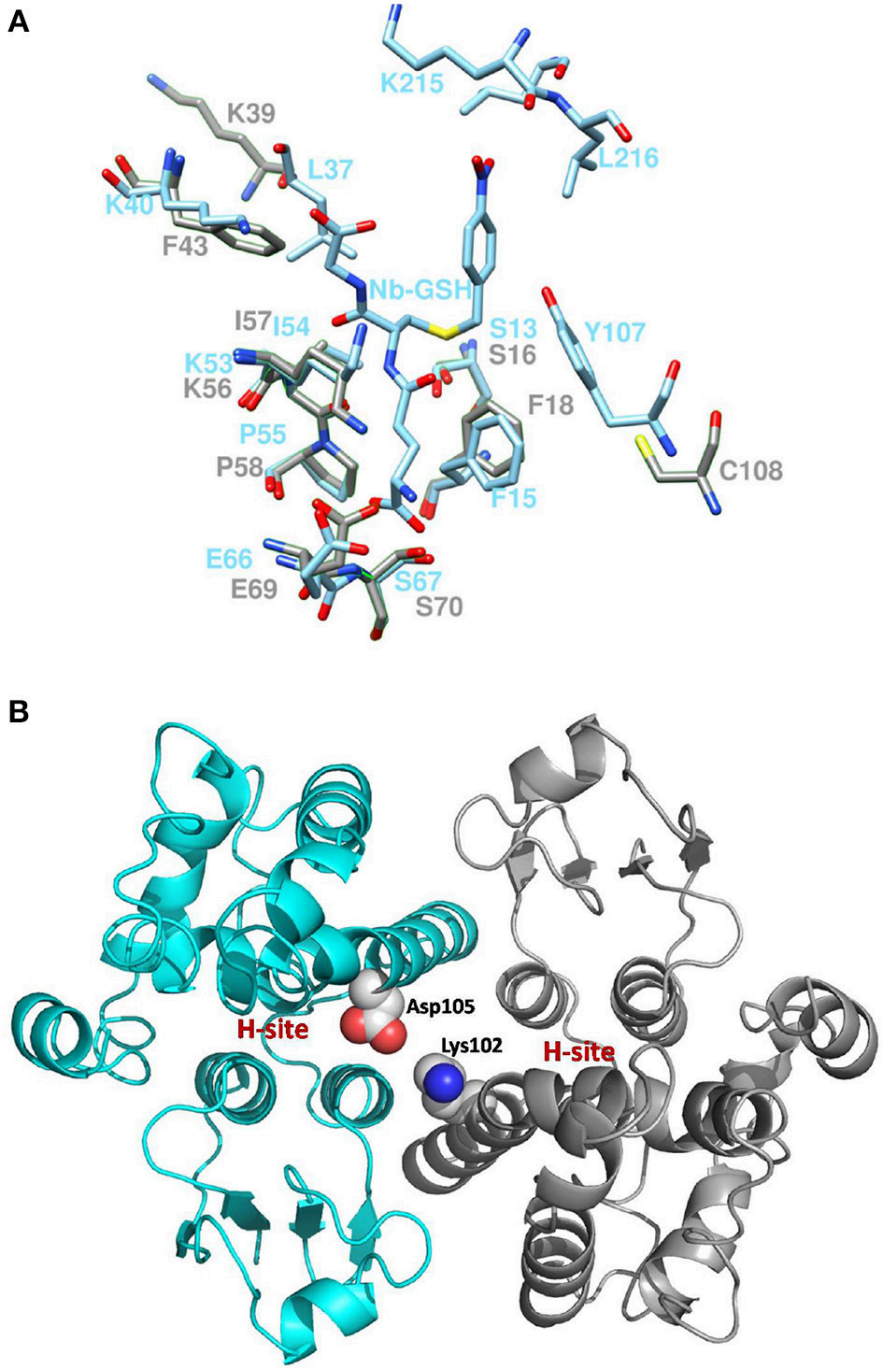
Active site comparison and residue interaction **(A)**. Comparison of *PvGm*GSTUG and *Gm*GSTU4-4 active sites after superposition. *PvGm*GSTUG and *Gm*GSTU4-4 residues are depicted and labeled in cyan and gray, respectively. Nb-GSH bound to *Gm*GSTU4-4 is shown **(B)**. Representation of the interaction between Asp105 and Lys102 in the *PvGm*GSTUG dimer. Asp105 and Lys102 are shown as spheres and are colored according to the atom type. The picture also depicts the location of the H-site in each subunit.

Coulombic surface analysis has previously shown that the G-site exhibits positive electrostatic potential, which may play a key role in -SH ionization of the bound GSH (Labrou et al., 2001). Similarly, the contribution of positively-charged residues in the adjustment of the electrostatic field has also been found in other GSTs (Patskovsky et al., 2000; Chronopoulou et al., 2012a). It is widely accepted that a Ser residue is the catalytic amino acid in GSTs of tau and phi classes (Labrou et al., 2001; Chronopoulou et al., 2012a), and that it stabilizes the deprotonated form (GS^−^) of bound GSH (Lo Piero et al., 2009). Structure superposition of *PvGm*GSTUG with *G. max* GSTU4-4 (PDB id 2vo4) revealed an rms deviation of 0.92 Å in Cα positions for 131 aligned residues and identified Ser16 as the catalytic residue. However, several changes were found in the vicinity of the active site (Figure 9). The conserved Glu69 and Ser70 correspond to Glu66 and Ser67 that form hydrogen bonds with the γ-Glu moiety of GSH (Figure 10A). The glycyl moiety of GSH interacts with Lys40 in *Gm*GSTU4-4. In *PvGm*GSTUG, a Phe residue replaces Lys, a change that could affect the orientation of GSH. Arg18, another conserved residue among tau GST sequences corresponds to Arg21in *PvGm*GSTUG. Arg18 has been suggested to stabilize the interactions between helices H1 and H4 through a strong electrostatic interaction with Asp103. A similar interaction appears also in *PvGm*GSTUG with Asp105, the structural equivalent residue of Asp103 in *Gm*GSTU4-4. Tyr107, a key residue at the H-site of *Gm*GSTU4-4, which forms aromatic interactions with the benzyl group of Nb-GSH in the *Gm*GSTU4-4–Nb-GSH complex (Axarli et al., 2009a). In *PvGm*GSTUG, it is replaced by a Cys residue, a change that could make the H-site more open and possibly alter its binding properties.

The subunit-subunit interactions in the folded dimeric structure of GSTs are important for both the stabilization of the tertiary structures of the folded subunits of the dimer as well as for the catalytic activity and substrate specificity. Comparison of the subunit-subunit interface revealed conservation of the interactions between the two subunits and of the hydrophobic interactions. *PvGm*GSTUG Val53 corresponds to Val50 in *Gm*GSTU4-4 and forms a lock with aromatic residues Phe99 (Phe97), Trp100 (Trp98), and Phe103 (Tyr101). A fourth hydrophobic residue, Leu134, is replaced by Ala134 in *PvGm*GSTUG, a change that may contribute to weakening of the interface. Salt bridges between Glu79 and side chains of Arg94′ and Arg98′ from the second subunit of the dimer are maintained as in *Gm*GSTU4-4 (Glu76, Arg92′ and Arg96′, respectively, in *Gm*GSTU4-4). Further analysis of the subunit-subunit interface revealed a putative mechanism that may affect the inter-subunit communication and promote the observed positive cooperativity. Structural examination revealed that the key residue bridging the dimer interface, Asp105, may play an important role in inter-subunit communication (Figure 10B). This residue could interact with Lys102 from the second subunit, forming a strong salt bridge. Since Lys102 is located in the α-helix H4, the signal may be transmitted via the α-helix H4 to the H-site residues (e.g., Phe117, Leu109), which are located at the end of this helix.

## Conclusions

We report here the first directed-evolution study of GST genes from different plants and provide the first crystal structure of a synthetic GST. The data demonstrate the power of protein engineering and DNA shuffling in developing enzymes with engineered catalytic activities. From the evolutionary point of view, the results show that the recombination of segments from homologous GSTs from different plants can generate synthetic enzymes of practical significance that can be exploited for the creation of more sustainable and environmentally-friendly biocatalysts. The unusual low K_m_ value obtained for GSH with CuOOH suggests that the enzyme is able to perform efficient catalysis under conditions where the concentration of GSH is low, such as in the case of oxidative stress. This supports the potential for the future application of this enzyme as a genetic tool in agricultural biotechnology for the development of genetically engineered plants with high resistant to stress conditions.

## Materials and methods

### Materials

All enzyme substrates and antibiotics were obtained from Sigma-Aldrich (USA). The pCR T7/CT-TOPO kit, pEXP5-CT TOPO TA Cloning Kit, DNAse I, and SuperScript^TM^ II reverse transcriptase were purchased from Invitrogen (USA). KAPA Taq and KAPA High fidelity DNA polymerase were purchased from KAPA Biosystems (USA). The miniplasmid isolation kit was purchased from Macherey–Nagel, (Germany). The QIAquick^TM^ Gel Extraction kit was purchased from Qiagen (USA).

### Methods

#### Plant growth and stress conditions

*P. vulgaris* and *G. max* seeds were pre-germinated (72 h at 30°C) on distilled water-moistened Whatman 2MM filter paper. After germination, they were transferred to plastic pots containing soil. The plants were grown in a controlled environment (12-h day/12-h night cycle, at 25°C during the day and 21°C during the night at 65% humidity) and watered with deionized water every 4 days. Plants (3–4 weeks after germination with three or four pairs of leaves) were stressed using a three-step protocol. In the first step, plants were sprayed with a mixture of heavy metals consisting of nickel (150 μM), zinc (200 μM), and chromium (50 μM) and left for 24 h. In the second step, a herbicide mixture composed of fluazifop-p-butyl (diluted 1:250), atrazine (0.2 mM), and alachlor (0.2 mM) in ethanol solution (20% v/v) was used to treat plants. In the third step, plants were subjected to heat stress at 37°C for 24 h. Control plants did not receive any treatment. Tissue samples (leaves, shoots, and roots) from treated and control plants were collected after 24 and 48 h.

#### GST activity measurements in *P. vulgaris* and *G. max* extracts in response to multiple stresses

For protein and GST enzyme assays, plant tissues (roots, shoots, leaves) of treated, and control plants were ground to a fine powder using a mortar and liquid nitrogen. The ground material was extracted with potassium phosphate buffer (50 mM, pH 6) containing 0.1 mM EDTA and 1% w/v polyvinylpyrrolidone (3:1 buffer volume/fresh weight. The homogenate was subsequently centrifuged at 13,000 × *g* for 10 min (4°C) and the supernatant was used for enzyme activity and protein determinations (Bradford, 1976), using bovine serum albumin as the standard. Enzyme activity was estimated toward CDNB, CuOOH, fluorodifen, ethacrynic acid, and p-nitrobenzyl chloride (Tappel, 1978; Satoh, 1995; Dixon et al., 2003; Axarli et al., 2009a).

#### Molecular cloning

Total RNA from leaves, shoots, and roots was isolated as previously described (Brusslan and Tobin, 1992) and checked by agarose electrophoresis for its integrity. Total RNA was subjected to DNase treatment with the RNase-free DNase. cDNA synthesis was achieved in a total volume of 20 μL using 1–5 μg of total RNA, 0.5 μg oligo(dT)12–18, 10 mM of each dNTP, and sterile water to a final volume of 12 μL. After incubation at 65°C for 5 min, 5 × superscript buffer, 10 mM dithiothreitol, 40 Units RNAseOUT^TM^, and 200 Units reverse transcriptase Superscript II (Invitrogen) were added in a thermocycler, which was operated at 42°C for 50 min and then at 70°C for 15 min.

Amplification of the GST genes by gradient PCR was performed using KapaTaq DNA polymerase and degenerate primers. Degenerated primers (Supplementary Table 1) were used in order to recover known and probably unknown GST sequences from *P. vulgaris* and *G. max*. The degenerated primers were designed based on nucleotide and aminoacid sequence alignments (Lang and Orgogozo, 2012) of theta class GST genes, derived from multiple related species (Axarli et al., 2009a; Han et al., 2018). The primers were designed based on similarities of the nucleotides at the 5′ and 3′ end sequences.

The following conditions were used for all sets of primers (see below) in a PCR volume of 50 μL: 1 μg cDNA, 10 pmol of forward primer, 30 pmol of reverse primer, 100 μM of each dNTP, 5 × KapaTaq buffer, and 1 Unit KAPA Taq DNA polymerase. The program used in the thermocycler was the same for all set of primers: 94°C for 60 s, T_m_ annealing 37°C for 90 s (the first 7–10 cycles), 44°C for 90 s (the next 7–10 cycles), 53°C for 90 s (the last 30–40 cycles), and 72°C for 50 s.

The PCR products were analyzed on a 1% (w/w) agarose gel and the corresponding bands were cut out and cleaned using the QIAquick^TM^ Gel Extraction kit (Qiagen), according to the manufacturer's instructions. The clean PCR products were A-tailed using Taq polymerase before being ligated to the pEXP5-CT vector using the TOPO®TA Kit (Invitrogen, USA). The recombinant plasmids (pEXP5-CT-GSTs) were used to transform competent *Escherichia coli* TOP10 cells.

#### Preparation of DNA for shuffling and construction of GST gene library

Recombinant plasmids (pEXP5-CT-GSTs) were mixed 1:1 in a final volume of 24 μL. The mixture was equilibrated at 15°C and supplemented with 5 μL of DNase buffer (400 mM Tris-HCl pH 8.0, 100 mM MgSO_4_, and 10 mM CaCl_2_), 21 μL of TE buffer (10 mM Tris pH 8.3 and 1 mM EDTA), and DNase (0.7 Units). At different times, aliquots of 6 μL were obtained and stop solution (20 mM EGTA, pH 8.0) was added and heated at 65°C for 10 min. Agarose gel electrophoresis 2% (w/w) of the DNase products was performed to check for digestion. Random fragments of 50–100 bp obtained after 8–15 min were selected for the shuffling procedure.

Reassembly of DNA fragments was carried out. The DNA fragments were used in PCR in the presence of 10 × Pfu buffer, 100 μM of each dNTP, and 1.25 units Pfu polymerase. The PCR cycle consisted of denaturation at 94°C for 0.5 min, annealing at 55°C for 1 min, and polymerization at 72°C for 1 65 s per cycle, with 40 repeats of the cycle to amplify the reassembled products. PCR reassembly product (1 μg) was used as template in a second PCR with the degenerate primers (Supplementary Table 1). This PCR contained 10 pmol of each forward primer, 30 pmol of each reverse primer, 10 × Pfu buffer, 100 μM of each dNTP, and 1.25 Units Taq/Pfu DNA polymerases. The reaction consisted of 11 cycles of denaturation at 94°C for 30 s, annealing at 55°C for 30 s, and polymerization at 72°C for 45 s, as well as of 14 cycles of denaturation at 94°C for 30 s, annealing at 55°C for 30 s, and polymerization at 72°C for 45 s and 25 s per cycle, followed by final extension of 10 min at 72°C. The product of this reaction was run on a 1% (w/v) agarose gel, excised, and purified using a QIAquick^TM^ Gel Extraction, kit (Qiagen). The extracted product was ligated to a T7 expression vector (pEXP5-CT/TOPO®TA). The resulting plasmid library was transformed into *E. coli* TOP10 and *E. coli* BL21(DE3) cells.

#### Screening of library, and expression and purification of recombinant enzymes

Screening of the library and expression of the recombinant enzymes were carried out as described by Axarli et al. (2016). Enzyme purification was carried out using affinity chromatography on S-hexyl-GSH-Agarose as previously described (Axarli et al., 2009a). Protein purity was judged by SDS PAGE.

#### Assay of enzyme activity and kinetic analysis

Enzyme assays were carried out as previously described (Axarli et al., 2009a; Skopelitou et al., 2012). The Bradford assay was used for protein determination. Kinetic analysis was performed as described by Axarli et al. (2016).

#### Thermal stability and inactivation

Thermal inactivation of purified *PvGm*GSTUG was performed in potassium phosphate buffer (20 mM, pH 7) for 5 min at different temperatures (15–65°C). The enzyme was subsequently assayed for residual activity (enzyme activity at 4°C was considered 100%). Melting temperatures (T_m_) were determined from the plot of relative inactivation (%) vs. temperature (°C). The T_m_ value corresponds to the temperature at which 50% of the initial enzyme activity is lost after heat treatment.

The thermal stability of *PvGm*GSTUG was also investigated using DSF on a Real-time PCR StepOne™ instrument (Applied Biosystems, USA). The thermal stability was measured in potassium phosphate buffer (20 mM, pH 7) using the Protein Thermal Shift™ Dye (Applied Biosystems, USA). Fluorescence monitoring was carried out at 10–95°C in increments of 1°C with a ramping rate of 2%. Melting temperatures (T_m_) were estimated using the Protein Thermal Shift™ Analysis Software (Applied Biosystems). Ligand-binding analysis was also achieved with DSF in the presence of different concentrations (0.1, 0.5, 1.0 mM) of GSH and S-(p-nitrobenzyl)-GSH under the same heating and buffer conditions.

#### Crystallization and data analyses

The protein was crystallized with the hanging drop vapor diffusion method using 2 μL of protein mixed with 2 μL of reservoir solution containing polyethylene glycol 4000, 20% (w/v), sodium succinate 0.2 M, and HEPES-NaOH (0.1 M, pH 7.0). X-ray diffraction data (Table 5) were collected on the ID23-1 beamline at the European Synchotron Radiation Facility (France) under cryogenic conditions (100 K). Crystals were initially transferred to a reservoir solution containing 20% v/v glycerol for 2 s and then flash-cooled in liquid nitrogen. Diffraction data were processed with XDS (Kabsch, 2010) and scaled with AIMLESS (Evans and Murshudov, 2013).

#### Structure determination, refinement, and analysis

Structure determination was pursued with the molecular replacement method using PHASER (McCoy et al., 2007). The structure of a *Ricinus communis* GST (PDB ID 4J2F; sequence identity 46.8% with *PvGm*GSTUG) was employed as the search model after modification with SCULPTOR (Bunkóczi and Read, 2011) that truncated side-chains from non-identical residues. Refinement was carried out initially with PHENIX (Adams et al., 2010) and subsequently with REFMAC (Murshudov et al., 2011). The low-resolution refinement options in REFMAC (Kovalevskiy et al., 2016) were utilized owing to the limited resolution of the structure. Structure-based sequence alignment was performed with Secondary Structure Matching (Krissinel and Henrick, 2004). The structure was validated using validation tools in COOT and PHENIX. Figures were created with CHIMERA (Pettersen et al., 2004). The structure has been deposited in the Protein Data Bank (PDB id 6GHF).

## Author contributions

NEL, EGC, and ACP conceived and designed the experiments; EGC and ACP performed the experiments; FA, PM, and IN-O analyzed the data; NEL, EGC, ACP, PM, and IN-O wrote the paper.

### Conflict of interest statement

The authors declare that the research was conducted in the absence of any commercial or financial relationships that could be construed as a potential conflict of interest. The reviewer AH is currently co-organizing a Research Topic with one of the authors NEL and confirms the absence of any other collaboration.

## Abbreviations

BDNB: 1-bromo-2,4-dinitrobenzene
CDNB: 1-chloro-2,4-dinitrobenzene
IDNB: 1-iodo-2, 4-dinitrobenzene
FDNB: 1-fluoro-2,4-dinitrobenzene
CuOOH: cumenehydroperoxide
DHAR: dehydroascorbate
Fluorodifen: 4-nitrophenyl 2-nitro-4-trifluoromethylphenyl ether
G-site: glutathione binding site
GSH: glutathione
GST: glutathione transferase
HED: 2- hydroxyethyldisulfide
H-site: hydrophobic binding site
*PvGm*GST: GST variant created by DNA shuffling of GSTome from *Phaseolus vulgaris* and *Glycine max*.
