# Supplementary material for: Expanding the Plant GSTome Through Directed Evolution: DNA Shuffling for the Generation of New Synthetic Enzymes With Engineered Catalytic and Binding Properties
Source: Front Plant Sci. 2018 Nov 30;9:1737. doi: 10.3389/fpls.2018.01737 (PMC6284010; doi:10.3389/fpls.2018.01737)
Supplement: Supplementary file 1 [file Table_1.docx]

**Supplementary Table 1. The primers that were used in the DNA shuffling method.**

| Plant1F | 5’ ATG GCW KCW AVT CAG GAR GAK GTG 3’ | T_m_=54^0^C/50 mM Na^+^ |
| --- | --- | --- |
| Plant1R | 5’ CTA TTT TGA AGC ARW RAG RCT TT 3’ | T_m_=46^0^C/50 mM Na^+^ |
| Plant2F | 5’ ATG RHR STG AAR GTR TAY GS 3’ | T_m_=44 ^0^C/50 mM Na^+^ |
| Plant2R | 5’ CTA RAY TGG AGS TSK GTA GAG YT 3’ | T_m_=52^0^C/50 mM Na^+^ |
| Plant3F | 5’ ATG RCW GAY GRG GTG GTT CT 3’ | T_m_=49^0^C/50 mM Na^+^ |
| Plant3R | 5’ CTA CTC DAT NWC NAA CTT CTT TCT 3’ | T_m_=49^0^C/50 mM Na^+^ |
| Plant4F | 5’ ATG GMN GAN GAR GTN GWN GWN ST 3’ | T_m_=50^0^C/50 mM Na^+^ |
| Plant4R | 5’ YTA NTT YTT NGC NWW NTT WAG 3’ | T_m_=40^0^C/50 mM Na^+^ |
