# Supplementary material for: Expanding the Plant GSTome Through Directed Evolution: DNA Shuffling for the Generation of New Synthetic Enzymes With Engineered Catalytic and Binding Properties
Source: Front Plant Sci. 2018 Nov 30;9:1737. doi: 10.3389/fpls.2018.01737 (PMC6284010; doi:10.3389/fpls.2018.01737)
Supplement: Supplementary file 2 [file Image_1.pdf]

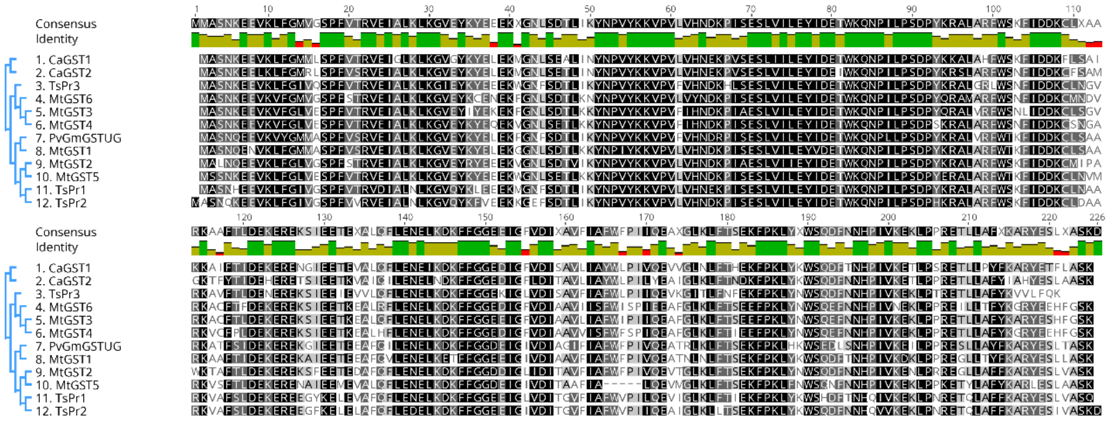

Supplementary Figure 1. Amino acid sequence alignments of *PvGm*GSTUG with the first 12 sequences identified following BLASTP search (Clustal Omega; Sievers et al., 2011) The figure was created using Geneious v9.1.2 software (Kearse et al., 2012) after alignment of the protein sequences using Clustal Omega (Sievers et al., 2011) and protein blast. Conserved areas are shown shaded: ■100% identity, ■80-100% identity, ■ 60-80% identity, □<60% identity. The order of the sequences in the alignment was ranked in the same order as obtained from the BLSTN and BLASTP searches. The accession numbers of protein sequences that were used were *Mt*GST.1 (*Medicago truncatula* GST, XP_003623196.1), *Mt*GST.2 (*Medicago truncatula* GST, XP_003623195.1), *Mt*GST.3 (*Medicago truncatula* GST, XP_003623174.1),*Ts*Pr1 (*Trifolium subterraneum* AU41833.1), *Ts*Pr2 (*Trifolium subterraneum* GAU41831.1), *Ca*GST1 (*Cicer arietinum* GST, ALZ41813.1), *Ca*GST2 (*Cicer arietinum* GST XP_004492376.1), MtGST.4 (*Medicago truncatula* GST, XP_013449023.1), *Mt*GST.5 (*Medicago truncatula* GST, XP_003623168.1), *Ts*Pr3 (*Trifolium subterraneum* GAU41828.1), and *Mt*GST.6 (*Medicago truncatula* GST, XP_003623173.1).
