# Supplementary material for: Expanding the Plant GSTome Through Directed Evolution: DNA Shuffling for the Generation of New Synthetic Enzymes With Engineered Catalytic and Binding Properties
Source: Front Plant Sci. 2018 Nov 30;9:1737. doi: 10.3389/fpls.2018.01737 (PMC6284010; doi:10.3389/fpls.2018.01737)
Supplement: Supplementary file 3 [file Image_2.pdf]

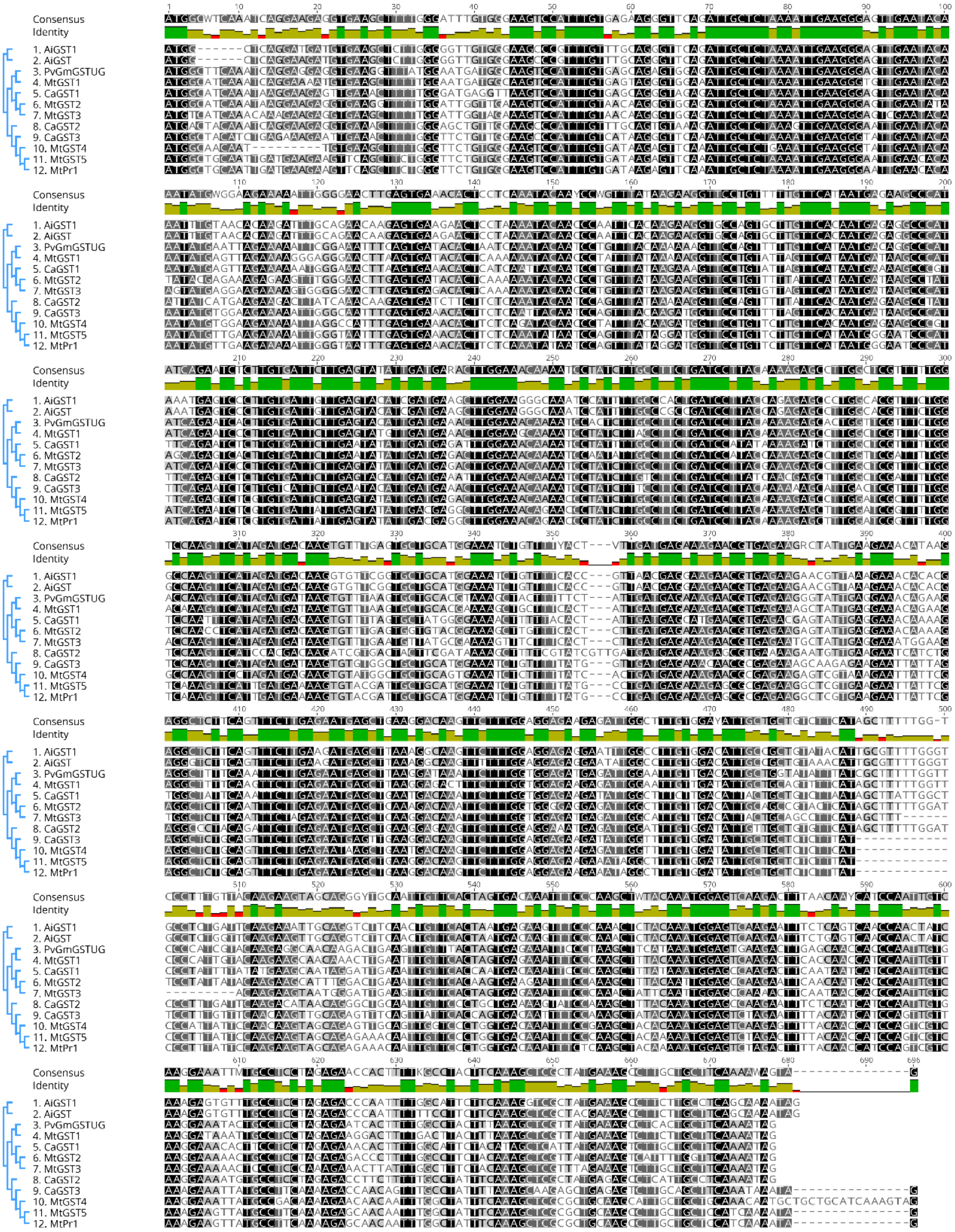

Supplementary Figure 2. **Nucleotide sequence alignments of *PvGm*GSTUG with the first 12 sequences identified following BLASTP search (Clustal Omega; Sievers et al., 2011).** The figure was created using Geneious v9.1.2 software (Kearse et al., 2012). Conserved areas are shown shaded: ■100% identity, ■80-100% identity, ■ 60-80% identity, <60% identity. The accession numbers of the sequences that were used were: *Mt*GST1, (*Medicago truncatula*, XM_003623148.2); *Ca*GST1,

(*Cicer arietinum,* XM_004492319.2); *MtGST2,* (*Medicago truncatula,* XM_003623126.2); *Mt*GST3, (*Medicago truncatula*, XM_003623120.2); *Ca*GST2, (*Cicer arietinum*: KT336759.1); *Ca*GST3, (*Cicer arietinum*, XM_012713550.1); *Mt*GST4, (*Medicago truncatula*, XM_003623159.1); *Mt*GST5, (*Medicago truncatula*5, XM_003623156.2); *Mt*Pr1, (*Medicago truncatula:* BT053471.1); *Ai*GST1 (*Arachis ipaensis*: XM_016342954.2); *Ai*GST2 (*Arachis ipaensis*: XM_016333558.2).
