# Supplementary material for: Expanding the Plant GSTome Through Directed Evolution: DNA Shuffling for the Generation of New Synthetic Enzymes With Engineered Catalytic and Binding Properties
Source: Front Plant Sci. 2018 Nov 30;9:1737. doi: 10.3389/fpls.2018.01737 (PMC6284010; doi:10.3389/fpls.2018.01737)
Supplement: Supplementary file 4 [file Image_3.pdf]

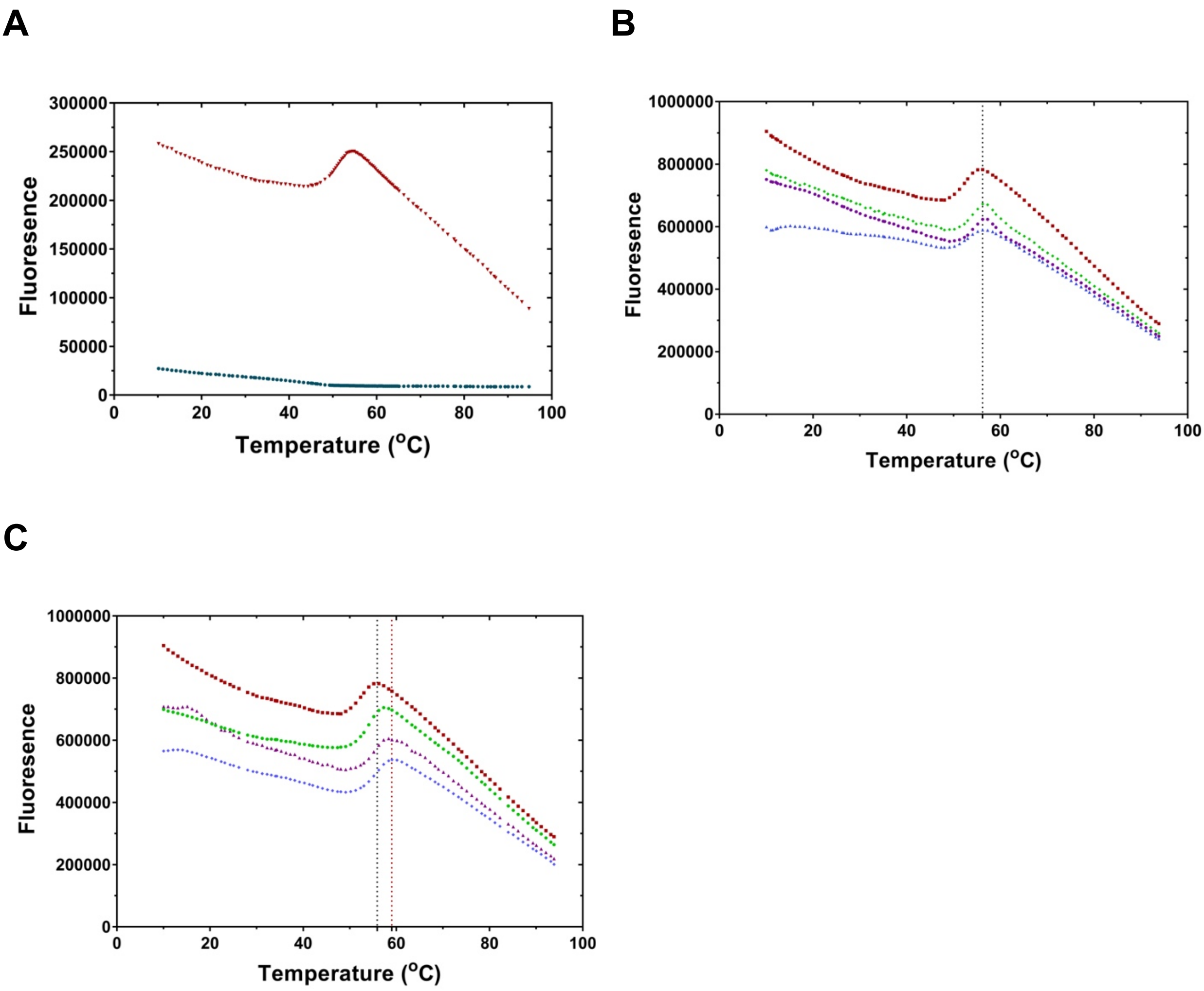

Supplementary Figure 3. Thermal inactivation curves for *PvGm*GSTUG A. Thermal denaturation curve (upper line) for *PvGm*GSTUG (1.43 µg) using differential scanning fluorimetry and response of control sample (lower line) **B**. Thermal denaturation curve for *PvGm*GSTUG (1.43 µg) in the absence (red line) or presence of GSH (green: 0.1 mM, purple: 0.5 mM, blue: 1 mM) using differential scanning fluorimetry. **C**. Thermal denaturation curve for *PvGm*GSTUG (1.43 µg) in the absence (red line) or presence of S-(p-nitrobenzyl)-GSH (green: 0.1 mM, purple: 0.5 mM, blue: 1 mM) using differential scanning fluorimetry.
